# Impact of scented candle use on indoor air quality and airborne microbiome

**DOI:** 10.1038/s41598-025-95010-0

**Published:** 2025-03-25

**Authors:** Hyunjun Yun, Ji Hoon Seo, Yong Gu Kim, Jinho Yang

**Affiliations:** 1 The AI Convergence Appliance Research Center, Korea Electronics Technology Institute, 226 Cheomdangwagi-ro, Buk-gu, Gwangju, 61011 Republic of Korea; 2 Department of Environmental Health, Korea University, 145, Anam-ro, Seongbuk-gu, Seoul, 02841 Republic of Korea; 3 Department of Occupational Health and Safety, Semyung University, 65 Semyung-ro, Jecheon, Chungcheongbuk-do 27136 Republic of Korea

**Keywords:** Indoor air quality, Scented candles, Particulate matter, Bacterial extracellular vesicles, Metagenomics, Environmental sciences, Air microbiology

## Abstract

Indoor air quality has become a growing concern worldwide due to its significant impact on human health, particularly in residential environments where people spend most of their time. Many studies have examined particulate matter (PM) in indoor air and indoor bioaerosols. However, there is a significant lack of research on airborne micro-sized bacteria (m-AB) and nano-sized bacterial extracellular vesicles (n-ABE), particularly those produced by common household activities, such as burning scented candles. This study investigates changes in PM concentrations and indoor microbiome composition resulting from candle use. Air samples were collected from three locations in residential homes: at the candle-lit spot (CL), 3 m away (3m_CL), and 6 m away (6m_CL). PM_10_ concentrations peaked at 1.52 times the baseline at the source after 5 min of burning, while PM_2.5_ and PM_1_ remained elevated at 3m_CL and 6m_CL over time. Before burning, dominant m-AB genera included *Phyllobacterium* and *Pseudomonas*, while post-burning, *Phyllobacterium myrsinacearum* in n-ABE significantly increased, marking its first detection in indoor air. This suggests that existing airborne bacteria may undergo growth or apoptosis due to combustion byproducts. These findings highlight the importance of improving ventilation in indoor spaces to minimize health risks from prolonged exposure to airborne particles and bacterial vesicles.

**Supplementary Information:**

The online version contains supplementary material available at 10.1038/s41598-025-95010-0.

## Introduction

Due to the lifestyle of modern society, most people tend to spend time in various indoor environments such as homes, job sites (workplaces), and transportation facilities^1^. In particular, urban residents spend approximately 90% of their time indoors, so their exposure to indoor pollution is gradually increasing^2,3^. The indoor submicron airborne particles concentration is also influenced by the infiltration of submicron airborne particle of outdoor origin into the indoor environment^4^. Additionally, harmful microbes, including bacteria, fungi, and viruses such as SARS-CoV-2, MERS-CoV, and SARS-CoV-1, have been identified as sources of submicron airborne particles^5–8^. Acute and chronic exposure to high concentrations of submicron airborne particles is associated with adverse health effects, including immune and reproductive disorders, infectious viral diseases, respiratory function impairments, and developmental issues^5,9–11^.

The use of scented candles is widespread for creating a pleasant home atmosphere, religious or spiritual reasons, odor removal, aesthetics, and therapeutic purposes^12^. However, scented candles, when using improperly refined wax materials, are known to emit particulate matter (PM), polycyclic aromatic hydrocarbons (PAH), and sulfur dioxide (SO_2_) during combustion^13^. Particularly, the concentration of fine particles generated during candle burning varies based on the combustion characteristics with investigations revealing the concentration compared to approximately 20 times higher under unstable burning conditions^14^. Another study reported low mass emission rates and particle diameters below 100 nm in “normal burning” mode, contrasting with high emission rates and particle diameters between 400 nm and 800 in the “soot” mode^15^. Measurements of indoor particulate matter (PM) resulting from candle combustion indicate that indoor PM concentrations can significantly exceed health-relevant standards. Studies have reported that PM_10_ (particles with an aerodynamic diameter ≤ 10 μm) concentrations from scented candle burning can reach up to 3.44 mg/m^2^, while PM_2.5_ (particles with an aerodynamic diameter ≤ 2.5 μm) levels can peak at 2.01 mg/m^2^^16^, far exceeding the Korean indoor air quality (IAQ) guidelines, which set 24-hour limits of 100 µg/m^3^ for PM_10_ and 50 µg/m^3^ for PM_2.5_^17^. Additionally, during stressed candle burning conditions, PM_2.5_ levels as high as 3.04 mg/m^2^ have been observed^18^. These findings suggest that candle use can contribute to increased indoor PM levels, often surpassing regulatory thresholds and potentially impacting indoor air quality.

In addition, scented candles have the potential to alter airborne microorganisms^19^. These alterations can be categorized into two types: bacterial cells (micro-sized airborne bacteria, m-AB) and bacterial extracellular vesicles (EVs) (nano-sized airborne bacterial EVs, n-ABE) released from bacterial cells. EVs are cell-to-cell communication materials that do not remain attached to the cell surface but detach and move to other cells. In the air, they can also detach from cell surfaces, remain suspended, and influence other cells^20^. Unlike whole bacterial cells, EVs are much smaller and remain suspended in the air independently of their parent bacterial cells, allowing them to be more easily transported through indoor environments^21,22^. Bacterial cells are generally micro-sized (typically larger than 500 nm), while bacterial EVs are nano-sized particles, typically smaller than 200 nm. Bacterial EVs function as cell-to-cell communication materials, containing nucleic acids, proteins, lipids, and metabolites^23,24^. Unlike whole bacterial cells, EVs can circulate through the bloodstream and directly interact with human cells, potentially impacting human health by modulating immune responses, influencing metabolic pathways, and facilitating the transport of bacterial components such as proteins, and nucleic acids. Studies have shown that bacterial EVs can enter the circulatory system even in healthy individuals, interacting primarily with monocytes and potentially reaching distant organs through systemic circulation. This suggests that despite their small mass, EVs can play a significant role in host-microbiota interactions, inflammation, and immune modulation^24–27^. Therefore, it is crucial to assess airborne bacteria and the airborne fractions of these bacterial m-AB and n-ABE to better understand their distinct effects on indoor air quality and health. However, most previous studies have not conducted this separation, limiting their insights into the health implications of nano-sized bacterial components.

While many studies have reported on the analysis of fine dust concentrations and the impact on human health resulting from the use of candles in most indoor environments^12,28–30^, there is limited evaluation and analysis of the impact of scented candles on submicron airborne particles and airborne microorganisms. Thus, the purpose of this study is to investigate the factors influencing submicron airborne particles and airborne bacteria, including m-AB and n-ABE, before and after candle burning in residential houses. This study’s results can provide essential parameters for future assessments of potential health impacts by evaluating the concentration and characteristics of submicron airborne particles and airborne bacteria due to candle combustion. Additionally, analyzing the correlation between indoor combustion and each pollutant can contribute to predicting the complex behavior of indoor pollutants, serving as useful data for setting guidelines or standards related to candle and combustion practices indoors.

## Results

### Alteration of particulate matter by lighting a scented candle

Sampling airborne particles was conducted at three locations in residential homes in Seoul, South Korea: the candle-lit spot (CL), 3 m from the source (3m_CL), and 6 m from the source (6m_CL). A blank sample (BL) was collected at the CL before burning the candle to establish a baseline.

**Fig. 1:**
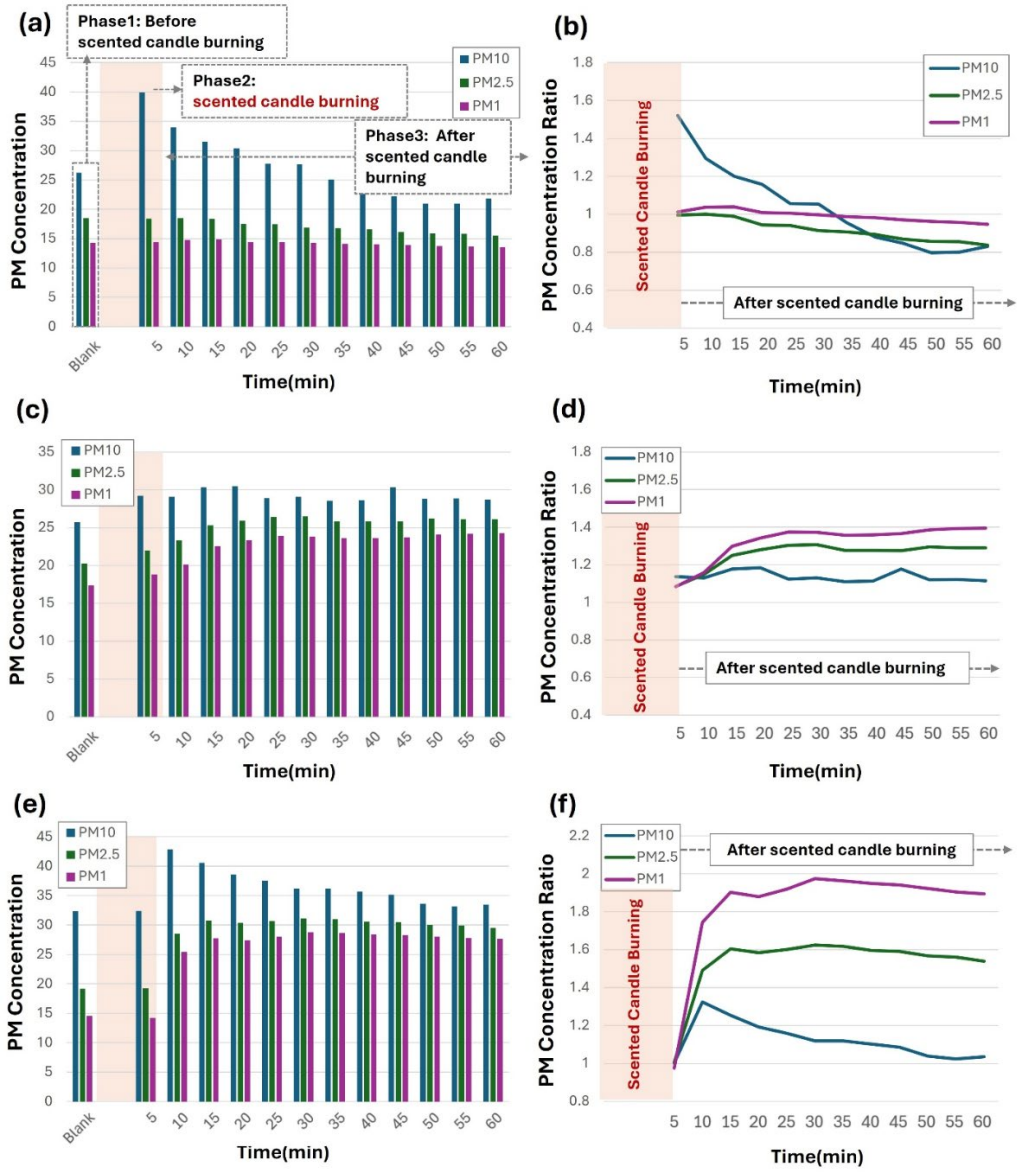
Alteration of particulate matter concentration by lighting a scented candle in places: (**a**) PM Concentration (CL), (**b**) Normalized PM Concentration (CL), (**c**) PM Concentration (3m_CL), (**d**) Normalized PM Concentration (3m_CL), (**e**) PM Concentration (6m_CL) and (**f**) Normalized PM Concentration (6m_CL). *Spot where scented candle was lit (CL), spot 3 m away from CL (3m_CL), and spot 6 m away from CL (6m_CL).

Figure 1a shows the changes in PM_10_, PM_2.5_, and PM_1_ (particles with an aerodynamic diameter ≤ 1 μm) concentrations at the CL during the before, during, and after burning phases. Five minutes after lighting, the PM_10_ concentration at the CL increased to 39.9 µg/m^3^, reaching a peak of 1.52 times the baseline before gradually decreasing to below the pre-burning level after 30 min. PM_2.5_ and PM_1_ concentrations increased by 0.1 µg/m^3^ and 0.5 µg/m^3^, respectively, and reached their maximum levels within 10 min before decreasing below baseline levels from 10 to 30 min onward. At 3m_CL, the PM concentrations followed a different trend, as shown in Fig. 1c. PM_10_ concentrations peaked at 42.8 µg/m^3^ after 10 min and remained consistently higher than the baseline throughout the burning phase. Meanwhile, PM_2.5_ and PM_1_ concentrations peaked at 31.1 µg/m^3^ (1.62-fold) and 28.8 µg/m^3^ (1.97-fold), respectively, at 30 min after burning. At 6m_CL, as presented in Fig. 1e, PM_10_ concentrations peaked at 30.5 µg/m^3^ after 20 min, representing an increase of 1.18 times compared to the baseline. PM_2.5_ concentrations reached a maximum of 26.5 µg/m^3^ (1.31-fold) after 30 min, while PM_1_ concentrations peaked at 23.9 µg/m^3^ (1.39-fold) at 25 min.

The normalized ratios presented in Fig. 1b and d, and f illustrate the temporal changes in PM_10_, PM_2.5_, and PM_1_ concentrations relative to the blank sample across the three sampling locations. At the CL, the PM_10_ ratio peaked at 1.52 after 5 min of burning, followed by a steady decline to 0.83 at 60 min. In contrast, the PM_2.5_ and PM_1_ ratios showed smaller fluctuations, with the PM₁ ratio stabilizing above 0.94 for most of the measurement period. At 3m_CL, the PM_10_ ratio remained close to the baseline in the early stages but increased to 1.53 at 60 min. PM_2.5_ and PM_1_ ratios increased more significantly, peaking at 1.62 and 1.98, respectively, after 30 min. These values indicate that finer particles dispersed more extensively over time compared to larger particles. At 6m_CL, the PM_10_ ratio peaked at 1.78 at 45 min before stabilizing at 1.11 at 60 min. PM_2.5_ and PM_1_ ratios at 6m_CL reached their maximum values of 1.39 and 1.39, respectively, at 25 min and remained elevated throughout the measurement period.

Figure S1 shows the ratio of PM_1_ and PM_2.5_ to PM_10_ at each time point during the burning phases across the three sampling locations. At the CL, the PM_2.5_/ PM_10_ ratio increased from 0.46 at 5 min to a peak of 0.76 at 50 min, while the PM_1_/ PM_10_ ratio increased from 0.36 to 0.66 over the same period. At 3m_CL, the PM_2.5_/ PM_10_ ratio ranged from 0.59 at 5 min to 0.90 at 55 min, and the PM_1_/PM_10_ ratio ranged from 0.43 to 0.84. Similarly, at 6m_CL, the PM_2.5_/ PM_10_ ratio started at 0.75 and peaked at 0.91 at 40 min, while the PM_1_/ PM_10_ ratio increased from 0.64 to 0.84 over time. These ratios indicate that fine particles (PM_1_ and PM_2.5_) made up a significant portion of total PM_10_ concentrations throughout the burning period, with the highest ratios observed at greater distances from the source.

**Fig. 2:**
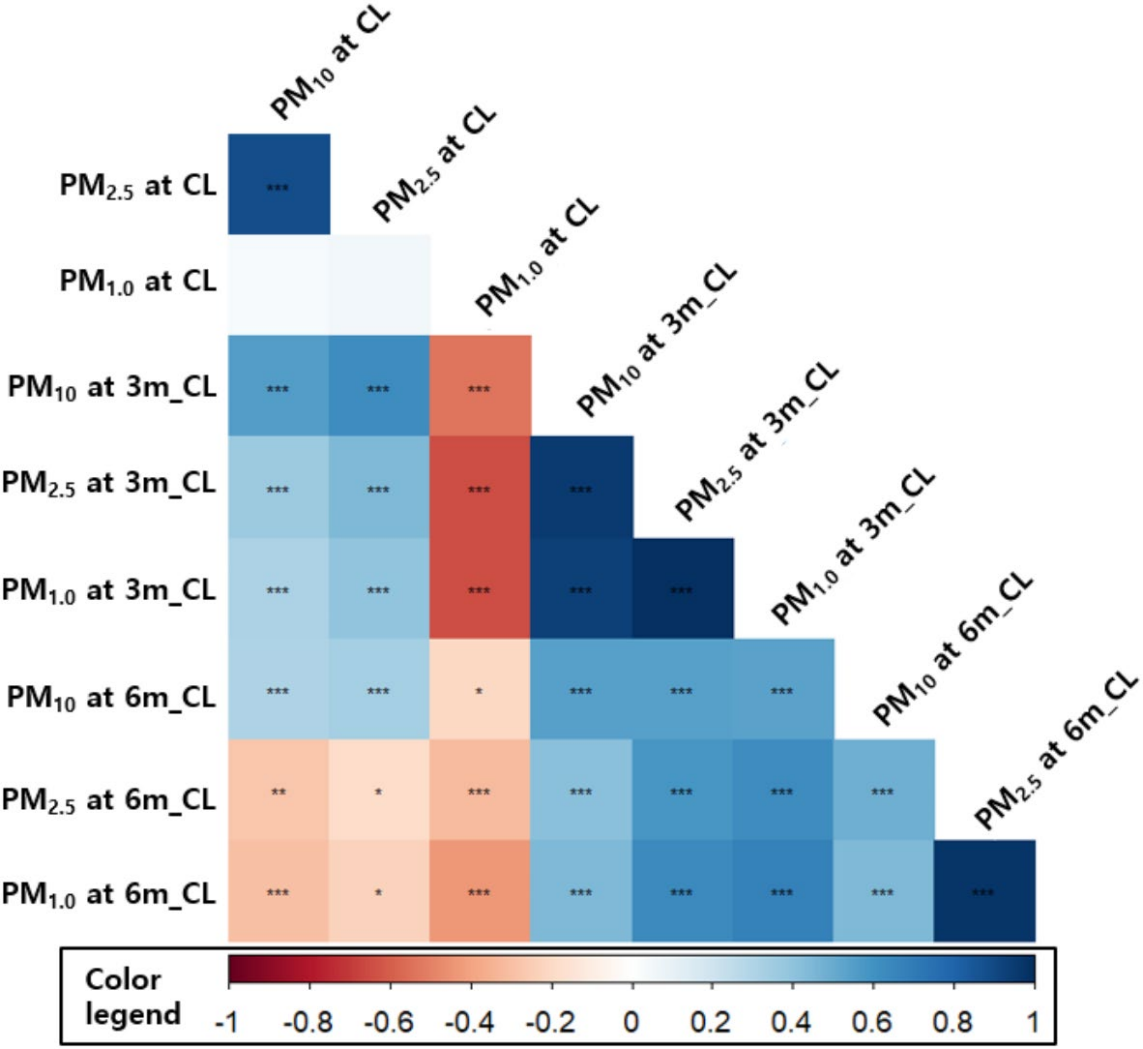
Correlation between particulate matter according to distance and size after lighting a scent candle (***: *p* < 0.001, **: *p* < 0.01, *: *p* < 0.05).

Figure 2 presents the correlation coefficients between PM sizes at the three sampling locations. At the CL, a strong correlation was observed between PM_10_ and PM_2.5_ (*r* > 0.88), suggesting that larger particles were emitted together near the source. However, the correlation with PM_1_ was weaker (*r* < 0.05), indicating a different dispersion behavior for finer particles. At the 3m_CL location, all correlation coefficients among PM sizes exceeded 0.94 (*p* < 0.001), indicating that particles of different sizes remain interrelated at this intermediate distance. At 6m_CL, the correlation between PM_10_ and PM_2.5_ weakened, with coefficients of 0.49 and 0.44, respectively, while PM_2.5_ and PM_1_ maintained a strong correlation of 0.98 (*p* < 0.001).

**Fig. 3:**
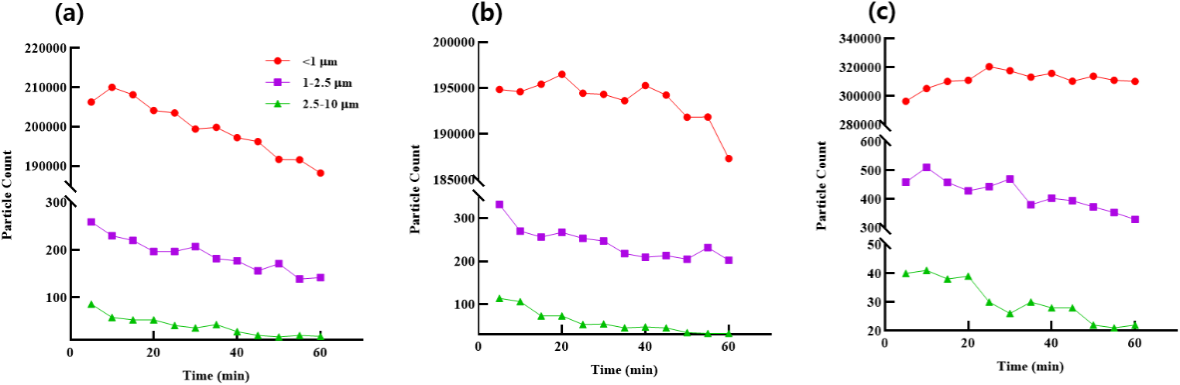
Particle number count by size range at different distances; particle number count over time categorized by size ranges. (**a**) CL (candle-lit spot), (**b**) 3m_CL (3 m from the source), and (**c**) 6m_CL (6 m from the source).

In addition to mass concentration analysis, Fig. 3 shows the number count of airborne particles across three size ranges: <1 μm, 1–2.5 μm, and 2.5–10 μm, measured over time at the three sampling locations. The number count of particles < 1 μm exhibited the highest values across all locations, particularly at 6m_CL, where concentrations consistently remained above 300,000 particles/cm^2^ during the first 30 min after lighting the candle. In contrast, the number count of larger particles (2.5–10 μm) rapidly decreased at all locations within the first 20 min, indicating gravitational settling of these particles.

At the CL and 3m_CL locations, the number count of particles < 1 μm remained relatively stable over time. However, a notable increase was observed at 6m_CL, suggesting that fine particles dispersed farther from the source and accumulated at greater distances. This trend aligns with the mass concentration results, indicating that finer particles are more likely to remain airborne and travel further in indoor environments during combustion events.

### Alteration of microbiological diversity

To analyze airborne bacteria based on particle size, we classified them into two distinct categories: m-AB and n-ABE. Specifically, m-AB refers to the 16 S rDNA extracted from micro-sized aerosol particles, whereas n-ABE refers to the 16 S rDNA associated with nano-sized bacterial EVs present in aerosols. The sum of m-AB and n-ABE thus represents the total airborne bacterial 16 S rDNA, which is typically referred to as airborne bacteria in previous environmental microbiology studies.

Alpha diversity indexes including observed species, Chao1, Shannon index, and Simpson index of m-AB and n-ABE for BL were lower than those in the other groups which were measured after lighting a scented candle. Observed species were highest at 3m_CL. In addition, Chao1 at 3m_CL of m-AB and n-ABE was higher than in the other groups, whereas Shannon and Simpson indexes at CL were higher than those in the other groups (Table 1).

**Table 1:** Alpha diversity of airborne bacteria samples (m-AB; micro-sized airborne bacteria, n-ABE; nano-sized airborne bacterial EV, CL; candle lit, BL; blank sample).

Alpha diversity		Observed species	Chao1	Shannon index	Simpson index
m-AB					
BL		14.0 ± 0.8	13.9 ± 0.01	0.57 ± 0.01	0.34 ± 0.01
CL		16.3 ± 3.1	17.6 ± 4.86	1.10 ± 0.47	0.46 ± 0.15
3m_CL		23.0 ± 3.3	24.7 ± 3.26	0.99 ± 0.05	0.36 ± 0.02
6m_CL		14.7 ± 3.1	14.3 ± 4.08	0.77 ± 0.03	0.35 ± 0.01
n-ABE					
BL		5.0 ± 0.8	11.0 ± 0.01	1.3 ± 0.01	0.57 ± 0.01
CL		11.3 ± 3.4	14.0 ± 4.83	1.96 ± 0.34	0.81 ± 0.05
3m_CL		14.3 ± 3.7	19.03 ± 4.83	1.76 ± 0.66	0.69 ± 0.15
6m_CL		11.7 ± 0.9	17.2 ± 2.48	1.89 ± 0.07	0.76 ± 0.05

### Metagenomic analysis of micro- and nano-sized airborne bacteria

**Fig. 4:**
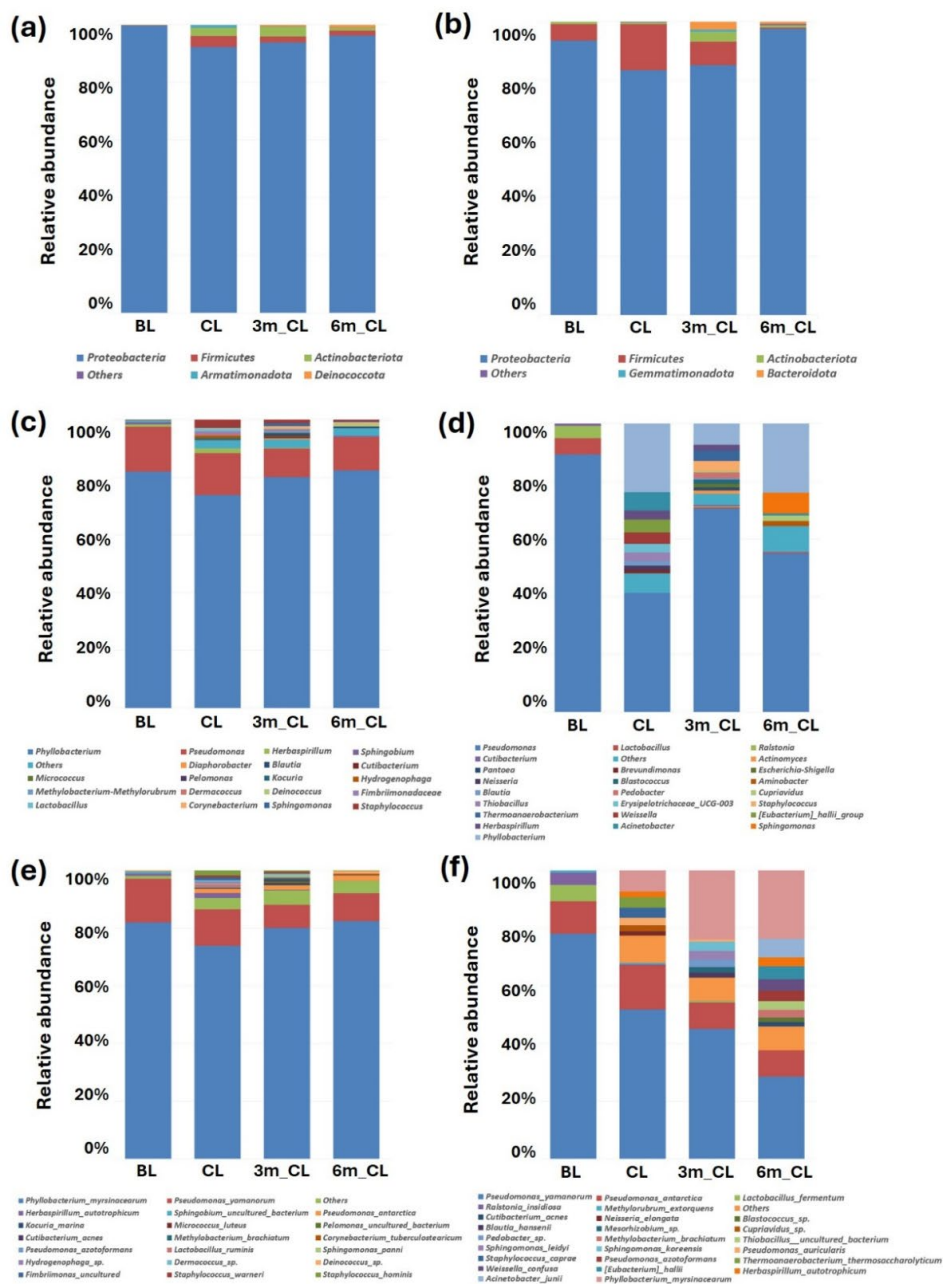
Averaged distribution of airborne microbiome according to pre- and post-lighting a scented candle. (**a**) m-AB at the phylum level, (**b**) n-ABE at the phylum level, (**c**) m-AB at the genus level, (**d**) n-ABE at the genus level, (**e**) m-AB at the species level, (**f**) n-ABE at the species level.

Dominant phyla of both m-AB and n-ABE was Proteobacteria, accounting for over 90% and 80%, respectively. Firmicutes of n-ABE were analyzed to account for more than 5% in BL, CL, and 3m_CL (Fig. 4a and b).

At the genus level of m-AB, *Phyllobacterium* was the most dominant genera with over 70% presence in all places, and *Pseudomonas* followed with 14.5 ± 5.0% at CL, 9.9 ± 4.5% at 3m_CL, and 11.5 ± 3.0% at 6m_CL. There were 13 genera in the air before lighting a scented candle, however, 35, 43, and 28 genera were analyzed at CL, 3m_CL, and 6m_CL, respectively (Fig. 4c). Although the number of observed genera at 3m_CL was higher than at other locations, this difference was primarily due to the detection of low-abundance genera (less than 0.1%). When excluding these minor contributions, the number of dominant genera remained relatively consistent across locations, with 23, 24, and 18 genera identified at 0m_CL, 3m_CL, and 6m_CL, respectively. However, at the genus of n-ABE, *Pseudomonas* showed the highest abundance in every sample, however, dominant genera were different between sampling places. *Lactobacillus* and *Ralstonia* were the dominant genera with 5.6 ± 0.5% and 4.3 ± 0.3%, respectively, before lighting a scented candle. After lighting a scented candle, *Phyllobacterium* increased dramatically to 23.8 ± 7.6% at CL, 7.4 ± 8.5% at 3m_CL, and 24.0 ± 7.3% at 6m_CL, respectively. *Acinetobacter*, *[Eubacterium]_hallii_group*, and *Weissella* were dominant at CL, *Thermoanaerobacterium* and *Staphylococcus* were dominant at 3m_CL, and *Sphingomonas* and *Methylobacterium-Methylorubrum* were dominant at 6m_CL. Additionally, there were 4 genera in the air before lighting a scented candle, however, 22, 25, and 21 genera were analyzed at CL, 3m_CL, and 6m_CL, respectively (Fig. 4d).

At the species of m-AB, *Phyllobacterium myrsinacearum* was dominant with over 73.8%, and the abundance of this species was not significantly changed. After lighting a candle, 33 new species including *Staphylococcus hominis*,* Staphylococcus warneri*,* Sphingomonas panni*, and *Lactobacillus ruminis* were identified at CL, 47 species including *Staphylococcus hominis*,* Staphylococcus warneri*,, *Sphingomonas panni*,* Methylobacterium brachiatum*, and *Cutibacterium acnes* species were identified at 3m_CL, and 29 species including *Deinococcus sp.*,* Corynebacterium tuberculostearicum*,* Ralstonia pickettii*, and *Sphingobium yanoikuyae* species were appeared at 6m_CL (Fig. 4e). On the other hand, at the species of n-ABE, after lighting a candle, composition of *Pseudomonas yamanorum*,* Lactobacillus fermentum*,* and Ralstonia insidiosa* decreased and *P. myrsinacearum* was identified, especially, this species increased dramatically at 0 m and 6 m sites. Additionally, 24 new species including *Acinetobacter junii*,* Herbaspirillum autotrophicum*,* [Eubacterium] hallii*, and *Pseudomonas azotoformans* species were identified at CL, 27 species including *Thermoanaerobacterium thermosaccharolyticum*,* Staphylococcus caprae*, and *Psedomonas auricularis* species were identified at 3m_CL, and 23 species including *Sphingomonas koreensis*,* Sphingomonas leidyi*, and *Methylobacterium brachiatum* species were identified at 6m_CL (Fig. 4f).

## Discussion

This study demonstrated that burning a scented candle significantly alters indoor air quality by increasing PM concentrations and influencing airborne microbial communities. Our findings showed distinct dispersion patterns of PM based on particle size, with PM_10_ concentrations peaking near the source (CL) and finer particles (PM₂.₅ and PM₁) dispersing further to 3m_CL and 6m_CL locations. These trends align with previous studies, which reported that larger particles settle more quickly due to gravitational forces, while finer particles remain airborne for longer periods and disperse farther^31,32^. Additionally, we observed that the number concentration of finer particles (PM_2.5_ and PM_1_) remained elevated even at the 6m_CL site, consistent with the Coanda effect, where warm air and particulates travel along ceilings and disperse into adjacent spaces^33,34^.

Furthermore, we found that burning a scented candle alters the airborne microbiome composition, particularly increasing n-ABE, such as *Phyllobacterium myrsinacearum*, which significantly increased after candle burning. This is a novel finding that has not been previously reported in indoor air studies. While previous studies on indoor microbiomes mainly focused on culture-based methods or mass concentration measurements, our study highlights the need to consider both microbial diversity and nano-sized particles, particularly bacterial EVs, which have potential implications for human health.

We conducted an experiment in a controlled environment with ventilation minimized to reduce external microbial influence. However, the complete exclusion of outdoor infiltration was not possible. Despite this, the observed microbial changes are likely primarily due to candle combustion, as external contributions were minimized. Additionally, combustion may promote microbial proliferation or apoptosis, leading to increased EV release. Previous studies have shown that indoor combustion events, such as cooking or smoking, can significantly alter airborne microbial communities through similar mechanisms^35,36^. These findings suggest that even low-intensity combustion events, such as candle burning, may influence indoor microbial diversity and warrant further investigation into their health implications.

Our study identified distinct dispersion patterns of PM based on particle size. Larger particles (PM_10_) exhibited higher concentrations near the source (CL) and decreased rapidly over time due to gravitational settling, whereas finer particles (PM_2.5_ and PM_1_) dispersed more extensively and remained elevated at greater distances (3m_CL and 6m_CL). This persistent increase in PM_2.5_ and PM_1_ concentrations at 3m_CL and 6m_CL highlights the potential for long-range dispersion of finer particles within indoor environments.

Previous studies have reported that more than half of the particles generated during candle combustion were smaller than 5 μm^37^. Our findings align with this observation, as we found that fine particles, including PM_2.5_ and PM_1_, dispersed more extensively and remained elevated at greater distances from the source. Additionally, Fine et al. noted that the diameter distribution of particles during candle burning tends to increase by over 50 nm^15^. Interestingly, it has been observed that more particles are generated during the extinguishing phase of candles than during the burning phase^38^, suggesting a significant influence of the soot mode^15^. Our results further indicate that soot particles can linger in the air for a considerable period, consistent with the findings of Cole et al., who reported that candle soot particles remain airborne within the 0.03 to 3 μm range^15,39^. This prolonged suspension of particles can lead to long-term exposure risks, particularly for ultrafine particles (UFPs) that penetrate deeply into the respiratory system. Moreover, the rapid ascent of high-temperature air and particulates, followed by lateral diffusion along the ceiling through the Coanda effect, explains the differential behavior of PM sizes at various distances from the source^40^.

In addition to mass concentration, we further analyzed the number count of particles across different size ranges to gain more insights into their dispersion behavior. The results showed that the number concentration of particles < 1 μm remained consistently high at the 6m_CL location, even after 30 min. This trend supports the hypothesis that fine particles are capable of remaining suspended in the air for prolonged periods and dispersing further from the source compared to larger particles. Conversely, particles in the 2.5–10 μm range showed a sharp decrease in number count over time across all locations, likely due to gravitational settling. These findings highlight the importance of considering both mass and number count in indoor air quality assessments, particularly for UFPs that pose greater risks to human health due to their ability to penetrate deep into the respiratory system^41,42^.

Yu et al. showed that a significant temperature increase of up to 6.3℃ at a height of 1.7 m indoors was observed during cooking with a stove, with a subsequent sharp increase in fine dust concentration observed in the adjacent room approximately 6 min after cooking^43^. Our findings corroborate these observations. Additionally, a pattern was observed where PM concentrations decreased at the 3-meter point but increased at the 6-meter point. Previous studies, such as those by Hooff et al., indicate that as smoke travels along the ceiling due to the Coanda effect, turbulence increases under low slot Reynolds numbers, leading to reduced clarity in identifying large-scale vortex structures in the outer regions of the jet. This finding suggests that PM concentrations may vary depending on distance and environmental conditions^44^. This study showed that lighting a scented candle increases the richness and evenness of n-ABE and stimulates activity of microorganisms. Heat events are related to the activity of microorganisms and microbial community^45^. For example, *Pseudomonas aeruginosa* increases at high temperatures, while the survival of *Bacteroides spp.* is higher at low temperatures than at high temperatures^22^. Therefore, the influence of heat can be negative or positive on microbial diversity. In addition, air pollutants had negative correlation with microbial biodiversity^46^. Candles, particularly scented ones, are known to produce complex pollutants depending on the characteristics of their flames^47,48^. Based on these findings, the extent to which microbial diversity is impacted will be further investigated in subsequent studies to determine the correlation. We suggest that the heat and air pollutants continuously generated by candles may have had some effect on microbial diversity.

In this study, airborne microbiomes were analyzed before and after lighting a scented candle. Before lighting a scented candle, we analyzed the dominant genera including *Phyllobacterium* and *Pseudomonas* of m-AB and *Pseudomonas*,* Lactobacillus*, and *Ralstonia* of n-ABE. On the other hand, previous studies reported that *Methylobacterium*,* Streptophyta_uncultured*,* Enterobacteriaceae_uncultred*,* Exiguobacteirum*,* Bacteroides*, *Micrococcus*,* Paracoccus*,* Staphylococcus*, and *Enhydrobacter* were dominant m-AB in indoor air at genus level^22,49^. In addition, *Methylobacterium*,* Streptophyta_uncultured*,* Enterobacteriaceae_uncultred*, and *Bacteroides* were dominant n-ABE in indoor air at genus level^22^. We suggest that the main reasons for the differences between this study and previous study were the lack of separation of microbial materials by size, the sampling environment such as air pollutants and thermal conditions, and methodology. Generally, genomic DNA isolated from samples includes micro-sized bacterial DNA and nano-sized bacterial DNA. In human stool sample, total bacterial DNA yield is composed of 72.9% micro-sized bacteria DNA yield and 27.1% nano-sized bacterial EV DNA yield^50^.

Additionally, after lighting a scented candle, airborne microbiome was changed in this study. For example, new species appeared, and the composition of species was changed. Air pollutants including PM_10_, PM_2.5_, and CO can affect the airborne microbial diversity and community^51^, and smoke such as wildfire and cigarette has also affected airborne microbiome^52^. However, there has been no research on the change of indoor environmental microbiota caused by a candle. Therefore, we suggested that atmospheric environments including thermal condition and air quality altered by lighting a scented candle might be main factors changing airborne microbiome. In particular, this study is the first to report the results that *P. myrsinacearum* EVs existed in air and increased dramatically by lighting a scented candle. Bacterial EVs was released by microbiome activity, therefore, the number of operational taxonomic units or species of EVs might be increased by active microorganisms such as through growth and apoptosis^22,53^. We observed that *P. myrsinacearum* was consistently dominant in m-AB both before and after burning the candle. However, in n-ABE, *P. myrsinacearum* exhibited a significant increase after the candle was lit. This indicates that the existing *P. myrsinacearum* in m-AB likely underwent growth or apoptosis due to the environmental changes induced by candle burning, leading to the release of EVs. Generally, *P. myrsinacearum* is known as gram-negative bacteria which was isolated from plant roots^54,55^. However, not much research has been conducted on *P. myrsinacearum*, and spondylodiscitis, an human infection, caused by *P. myrsinacearum*, was first reported recently^56^. However, there has been no previous study on the health effect of *P. mysinacearum*. Thus, we suggest that research on the human health effect and monitoring methodology of *P. myrsinacearum* which has the potential to be found in residential environments, should be conducted.

It was elucidated that UFPs can affect the human body directly through penetrating into bloodstream or blood circulation. UFPs enter the human body mainly through respiration and penetrate deep into the alveoli, and UFPs migrate to other organs such as the liver and kidney. The stay of UFPs in the lungs was less than that of PM_2.5,_ and UFPs remaining in immune cells in the lungs after inhalation were 8 times higher than PM_2.5_^57^. Additionally, oral administration of microbial EVs showed the systemic distribution throughout the whole body^53^. Therefore, nanosized particles including airborne UFP and bacterial EVs can have a more negative impact on human health, and previous studies have proved that indoor airborne bacterial EV is more important to human health than live bacteria. EVs derived from extracellular bacteria can lead to neutrophilic inflammation related to asthma, emphysema, chronic obstructive pulmonary disease (COPD), and lung cancer through a Th17 cell response. On the other hand, EVs derived from intracellular bacteria can cause mononuclear inflammation associated with the risk of emphysema through a Th1 cell response^24^. In addition, the environmental microbiome can cause changes in the human microbiome, and microbiome alteration is correlated with human diseases. In particular, EVs derived from human microbiomes have a greater correlation with human health. Previous studies have proved the important role of microbial EVs in the development and the pathogenesis of diseases, including skin, lung, and gut diseases, metabolic diseases, and cancers^23^. Therefore, we suggest that the alteration of environmental microbiome by events can be a significant factor for human health.

Although previous studies have analyzed airborne nano particles, this study was the first to analyze PM and bacterial EVs together. However, there were some limitations to this study. The first limitation was the number of sampling areas because the samples were collected from only three residential houses as a pilot study instead of collecting from various indoor facilities. Indoor systems, such as HVAC (heating, ventilation, and air conditioning) systems, air purifiers, sources, windows, furniture, the number of residents, interior structures, etc., differ between facilities. In addition, bioaerosols can be altered by meteorological conditions each day. Therefore, it is necessary to analyze airborne particles in various places. Furthermore, the type and scent of candles may also affect the composition of emitted particles and microbiomes. Therefore, it is necessary to conduct additional experiments using a variety of scented candles to determine how different candle types influence indoor air quality and microbial communities. The third limitation was the sampling method. In this study, to collect samples, we used a pump of 10 L/min with a gelatin filter. However, this method did not adequately represent bioaerosols because nano-sized aerosols float in the air and filter cannot collect all aerosols due to pore size and material. Thus, we suggest that standard sampling method for nano-sized aerosols is necessary. Finally, metagenomic analysis data showed only the distribution of microbiome. Taxa abundance data had a limitation in quantifying bacteria. We suggest that quantitative analysis data such as concentrations for bacterial EVs is necessary. Therefore, further studies should aim to sample from a wider variety of areas and use a more representative method. In addition, analyzing quantitative metagenomic data is necessary.

In conclusion, lighting a scented candle indoors can generate various size particles including airborne microbiome and small-sized particles that float in the air. Subsequently, indoor air quality and airborne microbiome are changed differently depending on the space. That is, lighting a scented candle can be related to human health. Therefore, it is essential to consider the duration and location of candle use and to manage airborne particles based on their size to reduce health risks.

## Methods

### Sampling and measurement

Sampling was performed on 3 spots at 3 residential houses, whose areas ranged from 26.5 m^2^ to 43.0 m^2^, in Seoul, South Korea: CL, 3m_CL, and 6m_CL. In addition, BL was collected at CL before a scented candle was lit. Air samples were collected using 37 mm gelatine membrane filter (Sartorius, Germany) for an hour with an air flow of 10 L/min at a height of 1.5 m. Natural ventilation was carried out for an hour before the sampling, and during the sampling, all of windows were closed and HVAC system was turned off. Filters were handled with sterilized tweezers and stored in sterile containers before and after the sampling to prevent contamination. Sampling equipment was cleaned and sterilized before each use. And then all of samples were stored at -20 °C.

In addition, the indoor PM concentrations, including PM_10_, PM_2.5_, and PM_1_, were measured using a Grimm portable aerosol spectrometer model 1.109 (Grimm Technologies, Inc., Douglasville, GA, USA) based on light scattering, with a flow rate of 1.2 L/min. Both particle number count and the corresponding mass concentrations were used for analysis. The scented candle was lit for 5 min and then extinguished, with measurements conducted for a total duration of one hour. Sampling was performed at three locations: CL, 3m_CL, and 6m_CL. At each location, measurements were taken during three phases: before lighting the candle, during the burning period, and after extinguishing the candle. The spectrometer was calibrated according to the manufacturer’s instructions prior to each sampling session to ensure accurate measurements.

### Metagenomic analysis of indoor airborne bacteria

Samples were diluted in 10 mL of phosphate buffered saline for 24 h. Pellet and supernatant containing cells and EVs in the samples, respectively, were separated by centrifugation at 10,000 × g for 10 min at 4 °C, a speed selected to minimize potential bacterial cell damage, as previous studies indicate that significant bacterial cell disruption occurs at ≥ 10,000 × g^58,59^. To further eliminate intact bacterial cells and foreign particles from the supernatant, a 0.22 μm filter was used. The pellet contained micro-sized bacterial cells, while the supernatant was enriched with nano-sized bacterial EVs. Metagenomic analysis was performed as in previous studies^22^. To extract DNA from the pellets and supernatant, filtered samples were boiled and centrifugated. DNA contained within the pellets and supernatant was extracted using a DNeasy PowerSoil kit (QIAGEN, Germany). The DNA extracted from the isolated bacterial cells and EVs contained in each sample was quantified using QIAxpert system (QIAGEN, Germany). Bacterial genomic DNA targeting 16 S V3-V4 hypervariable regions was amplified with V3 forward and V4 reverse primers as previously described^60^. The library preparation was given using PCR products, and each amplicon was sequenced using MiSeq (Illumina, USA). Taxonomy was assigned based on SILVA database 138 as previously described^60^.

### Statistical analysis

To avoid potential bias caused by differing sequencing depths, samples were rarefied to a depth of 15,000 reads in both micro-sized bacteria and nano-sized bacterial EVs for subsequent analysis. The correlation between PM concentrations was analyzed using Spearman’s rank correlation coefficients. Findings were considered significant for *p* < 0.05. The alpha-diversity of microbial composition was measured using the Shannon index, Chao1 index, and Simpson index to compare species richness and evenness. All statistical analyses were carried out using R version 4.3.2.

## Electronic supplementary material

Below is the link to the electronic supplementary material.

Supplementary Material 1

## Data Availability

The datasets generated during and/or analysed during the current study are available in the Sequence Read Archive (SRA) repository, PRJNA1165442.
