# Supplementary material for: Impact of scented candle use on indoor air quality and airborne microbiome
Source: Sci Rep. 2025 Mar 25;15:10181. doi: 10.1038/s41598-025-95010-0 (PMC11933706; doi:10.1038/s41598-025-95010-0)
Supplement: Supplementary file 1 — Supplementary Material 1 [file 41598_2025_95010_MOESM1_ESM.docx]

**Supplementary Materials**

**Insight into the Alteration of Indoor Particulate Matter and Airborne Microbiome Generated by Scented Candles: Impact on Human Health Across Particle Sizes**

Hyunjun Yun^1,†^, Ji Hoon Seo^2,†^, Yong Gu Kim^3^, Jinho Yang^3,*^

^1^The AI Convergence Appliance Research Center, Korea Electronics Technology Institute, 226 Cheomdangwagi-ro, Buk-gu, Gwangju, 61011, Republic of Korea

^2^Department of Environmental Health, Korea University, 145, Anam-ro, Seongbuk-gu, Seoul 02841, Republic of Korea

^3^Department of Occupational Health and Safety, Semyung University, 65 Semyung-ro, Jecheon, Chungcheongbuk-do 27136, Republic of Korea

^†^These authors contributed equally to this work.

***Corresponding author**

Jinho Yang, Ph.D.

Department of Occupational Health and Safety, Semyung University,

65 Semyung-ro, Jecheon, Chungcheongbuk-do, Republic of Korea

Tel: +82-43-649-1692, Fax: +82-43-649-1777, E-mail: [iamjinho@semyung.ac.kr](mailto:iamjinho@semyung.ac.kr)


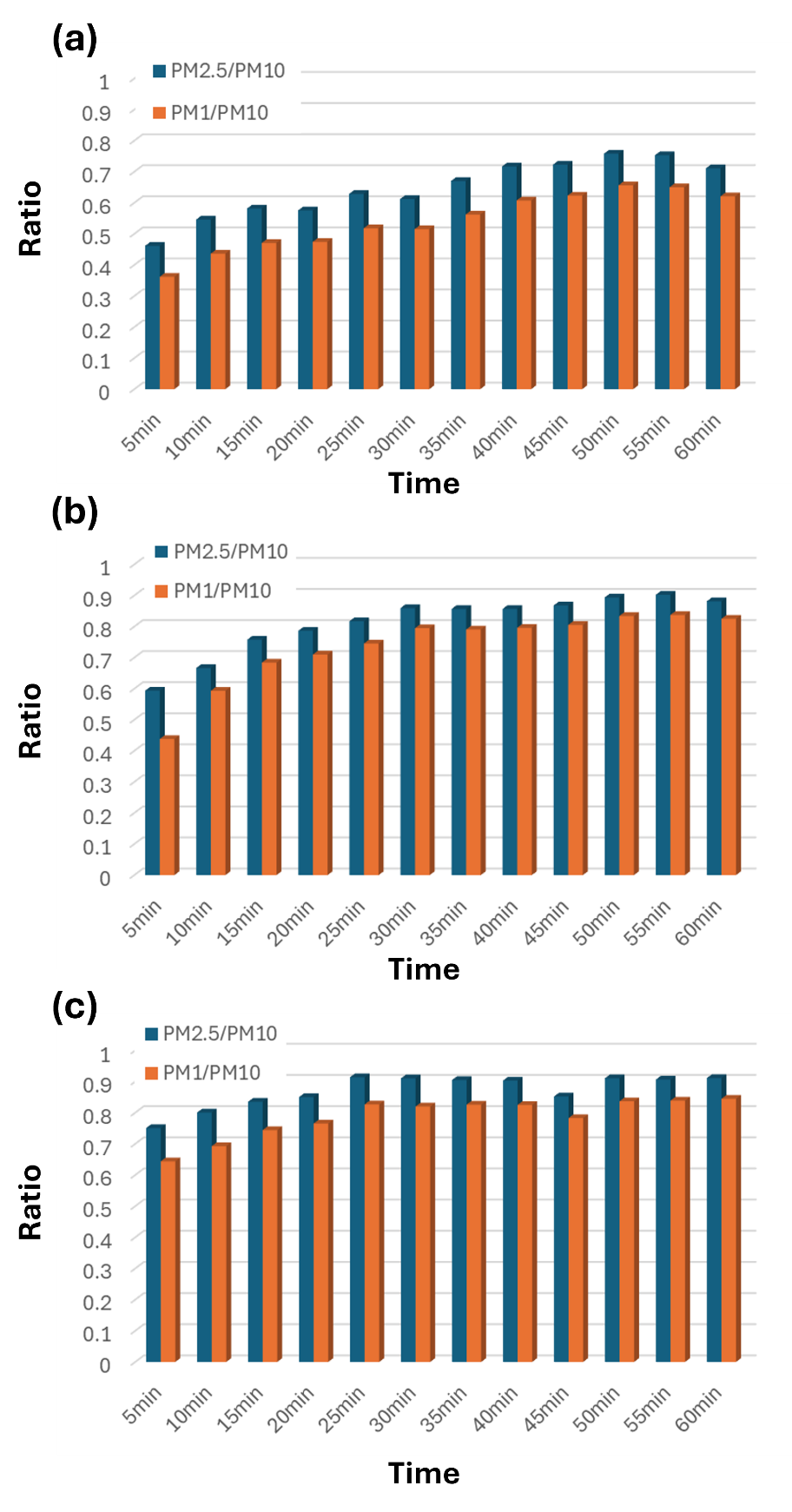


**Figure S1. Ratio of PM_2.5_/PM_10_ and PM_1_/PM_10_ over time at different sampling locations. (a) CL, (b) 3m_CL, (c) 6m_CL.**

*Spot where scented candle was lit (CL), spot 3m away from CL (3m_CL), and spot 6m away from CL (6m_CL)

| Product 1 | Product 2 |
| --- | --- |
| Paraffin wax, fragrance (*hexyl cinnamal*, etc.), allergenic substances (*hexyl cinnamal*, *butylphenyl methylpropional*, *benzyl salicylate*, *limonene*, *alpha-isomethyl ionone*), citronellol, coumarin | Paraffin wax, fragrance (*benzyl salicylate*, *hexyl cinnamal*), allergenic substances (*hexyl cinnamal*, *benzyl benzoate*), other components (*1,3,4,6,7,8-hexahydro-4,6,6,7,8,8-hexamethyl cyclopenta[g]-2-benzopyran*) |

**Table S1. Chemical Composition of Scented Candles Used in the Experiment**

Note:
*Detailed product information, including the manufacturer and specific product name, is not fully disclosed due to confidentiality concerns. However, this information can be provided upon request for experimental replication.*
